# eMedOffice: A web-based collaborative serious game for teaching optimal design of a medical practice

**DOI:** 10.1186/1472-6920-12-104

**Published:** 2012-10-31

**Authors:** Andreas Hannig, Nicole Kuth, Monika Özman, Stephan Jonas, Cord Spreckelsen

**Affiliations:** 1 Department of Medical Informatics, RWTH Aachen University, Pauwelsstraße 30, Aachen, 52074, Germany; 2 Division of General Medicine, RWTH Aachen University, Pauwelsstraße 30, Aachen, 52074, Germany; 3 Department of Diagnostic Radiology, Yale University School of Medicine, 333 Cedar Street, New Haven, CT 06520, USA

**Keywords:** Computer-Assisted Instruction/methods, Games, Experimental, Teaching/methods, Education, Medical, Undergraduate/methods, User-Computer Interface

## Abstract

**Background:**

Preparing medical students for the takeover or the start-up of a medical practice is an important challenge in Germany today. Therefore, this paper presents a computer-aided serious game (eMedOffice) developed and currently in use at the RWTH Aachen University Medical School. The game is part of the attempt to teach medical students the organizational and conceptual basics of the medical practice of a general practitioner in a problem-based learning environment. This paper introduces methods and concepts used to develop the serious game and describes the results of an evaluation of the game's application in curricular courses at the Medical School.

**Results:**

Results of the conducted evaluation gave evidence of a positive learning effect of the serious game. Educational supervisors observed strong collaboration among the players inspired by the competitive gaming aspects. In addition, an increase in willingness to learn and the exploration of new self-invented ideas were observed and valuable proposals for further prospective enhancements were elicited. A statistical analysis of the results of an evaluation provided a clear indication of the positive learning effect of the game. A usability questionnaire survey revealed a very good overall score of 4.07 (5=best, 1=worst).

**Conclusions:**

We consider web-based, collaborative serious games to be a promising means of improving medical education. The insights gained by the implementation of eMedOffice will promote the future development of more effective serious games for integration into curricular courses of the RWTH Aachen University Medical School.

## Background

The German healthcare system faces a downturn in the number of general practitioners starting or taking over a practice, especially in the rural areas [1,2]. Financial and administrative incentives have been introduced to improve the situation. In Germany most general practitioners own their medical practice or at least most of their equipment. They act as entrepreneurs and take the full economic risk. Recent reforms in the German healthcare system have granted patients more freedom to choose a general practitioner and, as a result, encourage competition [3,4]. Nonetheless, medical education is challenged to play its part by preparing medical students to cope with the increasingly risky and cost-intensive venture of a practice takeover or start-up. In the context of economics studies business games have been proven to support training in the skills necessary for starting up an enterprise [5,6]. Enhanced by computer-based communication and simulations, similar game-based learning interventions have evolved to a new category of serious games. Recently, serious games have been successfully applied to some fields of medical education [7,8]. The challenge of adequately preparing medical students for future practice start-up provides an excellent opportunity for further developing and testing collaborative aspects of serious games in medical education.

A general-purpose business game (or business simulation) has been defined as a complex man-made environment, where participants are able to gain experiences when acting in a simulated reality [9,10]. Yet there is open discussion concerning the definition of the term serious game. A relatively broad definition of a serious game is ‘any computerized game whose chief mission is not entertainment and all entertainment games which can be reapplied to a different mission other than entertainment’ [11]. In contrast to teacher-centred learning approaches, which primarily rely on the control and activity of the teacher, serious games are considered as inherently learner-centred and focusing on the needs of learners [12]. Experts in the field of serious games predict a continuous growth in this research area [13,14]. Recently, research activity in the field of serious games has indeed markedly increased and has especially addressed their application in medical environments [15]. Ritterfeld and colleagues (2009) found that 8% of all existing serious games deal with a health- or medicine-related topic [16]. Medical learners showed a high acceptance of serious games as shown in several studies: The INMEDEA simulator, which presents virtual case studies and adopts an open, nonlinear problem-oriented problem-solving approach situated in a virtual hospital, was judged as a good or very good teaching method by 76% of 70 test candidates [17]. As a second example the Junior Doctor Simulator (JDoc), an interactive, 3D, third-person view prototype focusing on the training of doctor-patient interactions, was considered to provide valuable experiences of patient interactions and routines of a hospital ward to junior doctors [18]. Evaluation results suggest a significant impact of serious games' stimulation of conceptual thinking on the learning outcome [19]. JDOC and INMEDEA teach common procedures of the everyday work of doctors in different contexts and focus on doctor-patient interaction and medical diagnosis. Recent projects have tried to close the gap between computer-supported learning and reality by introducing learning support to mobile devices [20,21]**]**. Improved collaboration induced by serious games in the nursing context was stressed some time ago [22]; in a more recent review of virtual 3-D medical learning environments the collaborative aspect was identified as a major strength associated with several game-oriented medical learning approaches based on virtual worlds [7]. Following the idea of applying a serious game approach to prepare medical students for future practice start-up the ‘Business Game: How to start a Medical Practice’ was set up as a joint project of the Division of General Medicine and the Department for Knowledge-based Systems of the Institute for Medical Informatics of the RWTH Aachen University Medical School. The learning scenario consists of different learning and computer-aided game phases covering an online search, rough and fine-grained planning of funding and the detailed planning of required medical equipment. A special computer-assisted module of the business game is the interactive, collaborative serious game eMedOffice. The game eMedOffice focuses on practical exercises guiding the participants towards strategies for an optimization of the interior design, workflows, equipment, components and furnishing arrangements of a medical practice. The goal of eMedOffice is to provide fun access to the necessary theoretical knowledge to promote relevant practical problem-solving skills of the learners. In this work we present the theoretical approach, technical realization, and evaluation results of eMedOffice that evidence the positive effect of this learning game.

## Methods

### Learning Design

**Figure 1:**
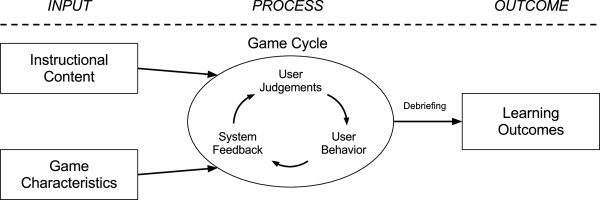
**Input-Process-Outcome Game Model is an inherent model of instructional games adapted from Garris and colleagues (2002) **[10].

**Figure 2:**
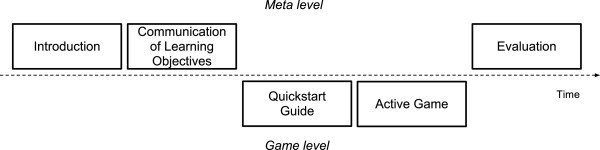
**Game phases of eMedOffice adapted from Ulrich (2006) **[29].

Research on business games yielded an abstract game model, which has been applied to the design of eMedOffice. The model includes the input, process and outcome objectives (Figure 1). As an input objective, the model integrates instructional content and specific game characteristics into eMedOffice. The input triggers a game cycle that consists of the user’s judgements and behaviour, and the system’s feedback. A variable number of iterations of this cycle during the process are possible. These iterations are similar to the cycle of expertise which describes the approach of experts to creating new strategies to solve problems [23]. The application of the cycle of expertise fosters a motivating gaming experience and encourages the exploration of self-invented ideas. Finally, the game cycle ends with a debriefing that evaluates the learning process and ensures a positive learning outcome. As seen from the perspective of the learner, the learning scenario contains a sequence of different learning phases (Figure 2): it starts and ends on a meta level introducing the game and evaluating the process. On the game level, the learners are involved in the preparation of the game and their individual gaming activities (e. g. understanding the games’ rules or choosing room functionalities). The described cycle of expertise resides in the active game phase. As game characteristics we adopted aspects of successful computer entertainment games like *challenge*, *control*, *fantasy*, *clear rules* and *clear objectives* in order to increase the willingness to learn. Some principles of good learning games serve as learning concepts of eMedOffice: *Empowered learners* (player is co-designer, active agent, with own identity in a customizable game play), *problem-solving* (problems are well-ordered and information is given when needed), and *understanding* (skills and strategies to be learned are embedded into a larger context and acquired by learning through experience) [24]. Each of these principles consists of more sub-principles. One example of a sub-principle for empowered learners is that learners feel like active agents and not just like passive consumers; another example is the cycle of expertise which is part of the problem-solving principle. 

### Knowledge acquisition, representation and maintenance

The game has been developed in close cooperation with medical students and the Division of General Medicine of RWTH Aachen University Medical School. In preliminary work, the implemented step-wise structure and learning objectives of the ‘Business Game: How to start a Medical Practice’ (eMedOffice) were acquired. Oriented on a business game used in economic sciences at RWTH Aachen University named EasyStartup, the requirements were analysed in several meetings with students and experts in medical practice design. Using a top-down approach, the game steps were iteratively refined to provide a convenient computer-supported way to achieve the learning objectives in an explorative manner. Acquisition of required special knowledge on how to characterize a medical practice for eMedOffice with sound workflows and efficient design was part of an on-going dissertation project (Rühr 2012, to be published). In particular, it was the main task of the dissertation to find rules usable for scoring and to verify the external validity of the scoring system. We used a pattern language that we tailored to our particular needs to gather and transfer the specific knowledge from the medical domain to an operational format. This acquired domain knowledge was represented and formalized in an ontology. A short and general definition of an ontology is given by Gruber and colleagues: 'An ontology is an explicit specification of a conceptualization’ [25]. The ontology editor Protégé was used for acquisition and maintenance of the semantic model [26]. We developed a transformation for the representation of non-branched workflows to concepts of an ontology that allowed a visual simulation of many common non-branched workflows of general practitioners. By using the A* path-finding algorithm we integrated a simulation of agents on the ground plan [27].

### Scoring algorithm

Conditions for a medical practice with sound workflows and adequate furnishing of a general practice are formalized as ontology restrictions. An evaluation algorithm verifies these restrictions by calculating an appropriate score. Examples of integrated rules formalized as restrictions are ‘All beds must be usable’ and ‘In the briefing room there is a medicine cabinet’. Many rules check the existence of furniture and equipment items in a specific room. Others check useful composition and usability of placed furniture and equipment items. A score is assigned to each of these rules. If a player’s solution meets the rule, the assigned score is added to the player’s total score. The score calculation is done by a conformity check of the restrictions with a student’s solution which is translated to an instance of the ontology-based object model. The result of this conformity check is a list of rules that the current student’s solution complies with and those with which it does not. A scoring algorithm calculates the final score based on the former rules according to a score table. For this calculation, we used a custom language developed for the purpose that is able to address the specific conditions of an ontology.

### Learning objectives

The learners should realize the fundamental relationship between the needs of patients and staff arising during typical processes in a medical practice on the one hand and the requirements concerning (a) the assignments of functions to rooms and (b) the arrangement of furniture and technical equipment on the other hand. The participants should gain orientation on the usage of rooms, furniture and equipment during the daily routine. Players should develop useful furniture and equipment compositions in the different rooms. In this game players do not learn factual knowledge by heart. Instead players are encouraged to explore self-invented solutions to problems when arranging a medical practice. The game is intended to teach observation and analytical skills as well as planning and deductive skills in the context of furnishing a medical practice by using immediate feedback of simulated patients. Thus, as a primary goal the participants should learn to react to patients’ (and staff's) problems caused by suboptimal arrangement of rooms, furniture items and equipment items. This goal reflects the fact that requirements may change dynamically over time. The participants should adopt a patient- and process-oriented approach to adjusting the organization of a medical practice.

### Hardware / software platform

Evaluations of the game used a virtual linux server machine with one gigabyte of RAM and 50 megabytes of free hard disk. For execution on the server side the game depends on common web techniques like a web server and a standard database that supports SQL. Clients of the game use a standard web browser without any requirements for extensions. To play eMedOffice, an active network connection between client and server is required. For a smooth visualization of integrated workflows the client should have at least a 1.0 GHz CPU. Thus, the hardware requirements do not exceed the standards of available computer hardware.

### Curricular context

As part of a revised six-year medical curriculum (‘Aachen Model Curriculum Medicine’), the game is embedded into the compulsory elective part (the so-called Individual Qualification Profile - IQP). The game is part of an IQP module addressing family medicine. Students may choose the module between the third and the sixth year. A group of 15 to 25 students regularly takes part in the module each semester.

### Evaluation design

We performed a quantitative summative evaluation consisting of two interventions. The evaluation was conducted in the compulsory elective ICP module 'family medicine' at the RWTH Aachen University Medical School during the four semesters between autumn 2009 and summer 2011. Thus, participants who took part in the IQP automatically participated in the evaluation. The number of participants varied from seven to 12 of mixed gender each semester. All participants attended both interventions. Each evaluation session took exactly 90 minutes. As computer techniques and online learning programs are integrated into many courses of the Medical School in Aachen, students are familiar with web-based techniques. Therefore, the participants were expected to deal with common computer handling and working with a web browser. The students sat next to each other and every participant had his/her own computer to play eMedOffice. Because of this constellation, all participants had the chance to look at the interim results of their immediate neighbours. A usability questionnaire for systematic determination of the quality of the serious game was the first intervention (see Appendix A, Appendix B). It was our intention to discover the answer to the central question ‘Does the quality of the serious game meet the requirements of a practical real-world learning application?’. To answer this question all participants of the first intervention had to fill in an evaluation sheet containing 22 scale items from five (best) to one (worst) after playing the serious game. We chose questions from an evaluation of learning software published by Holzinger in 2003 [28]. In order to test the consistency of results of this first intervention we computed an intra-class correlation coefficient (ICC) using absolute agreement of two-way random measures. By using 22 scale items in the usability questionnaire we were able to target specific assets of the serious game. One of the questions put to the target audience was ‘Is the software appropriate for the target audience?’. Another question addressed the loading time with ‘Is the loading time satisfactory?’. For an overview of all questions see Appendix A. Not all participants filled in the page of this first intervention because two classes used another page that was not compatible. As a second intervention, we conducted a self-report quantitative evaluation to find out if the serious game was able to support learning processes (see Appendix B). This intervention’s central question was: ‘Does the serious game help medical students to become familiar with the interior furnishings of a medical practice?’. Each participant had to fill in two self-report evaluation sheets covering the same seven items. The self-report used scale items from 5 (best) to 1 (worst). Table 1 lists the scale items for self-report. The first sheet had to be completed before the serious game could begin. Thus the first sheet represented a self-report (pre) of the knowledge of the participants without knowing the concrete learning content of the serious game. After the training, participants had to fill in the second sheet that covered two self-reports. One of those was a retrospective self-report (retro), so the participants had to self-assess their prior knowledge before the training again. The second self-report on the second sheet was an assessment (post) addressing their knowledge after playing the serious game. All three reports are integrated into the action sequence schedule of the learning session (Table 2). Each intervention was supervised by an educational adviser who had expert experience of the technical aspects of the serious game and basic knowledge on the medical information formalized in the game’s knowledge base. Besides the evaluation sheet, the oral feedback of the participants was recorded by the educational supervisor to provide an additional source of qualitative information for evaluation. For analysis of the second intervention the statistical method repeated measures analysis of variance (ANOVA) paired t-test (alpha = 0.05) was used. The null hypothesis states that no differences between the test groups exist and all groups are equal. 

**Table 1:** Self-report items

***St. no.***	***Statement***
1)	I am able to name important rooms
2)	I am able to name important furniture
3)	I am able to name important equipment
4)	I am able to estimate the financial outlay for interior furnishing
5)	I know the target rooms for placing furniture correctly
6)	I know how the furniture in specific rooms should be equipped
7)	I have an idea about good arrangements of furniture

The first column shows the *statement number* used as a reference to the different scale items. The second column *statement* names the used sentence for self-report.

**Table 2:** Timing chart table

***Phase***	***Action***	***Duration (min.)***
Introduction	Students arrive at the training room and sit down	5
	Educational supervisor greets students and gives an overview of the agenda	3
	Students fill in the first evaluation sheet self-report (pre)	3
	Educational supervisor collects first evaluation sheets	2
Execution	Educational supervisor gives brief instructions on how to start eMedOffice	3
	Students start web browser, navigate to the correct web address, register and log into eMedOffice	3
	Students read the introduction	5-10
	Students play eMedOffice	45
	Educational supervisor tells students to stop game play	1
Debriefing	Students stop playing and discuss eMedOffice results	5-10
Evaluation	Students fill in second evaluation sheet: self-report (retro and post)	3
	Students fill in usability questionnaire	5
	Educational supervisor collects second evaluation sheet as well as usability questionnaire and says goodbye to students	2
	Students leave the training room	1

The first column *phase* shows the phase of eMedOffice in which the action takes place. The column *action* details the planned action. The last column *duration* presents the estimated time in minutes for performing the planned action.

## Implementation

The game eMedOffice is an interactive, web-based, 2D, bird's eye view simulation of a medical practice prototype. Its purpose is to teach optimization of interior design, including the furnishing as well as equipment components of a medical practice of a general practitioner. It is playable in every standard web browser and hence available anytime at any location where an internet connection is accessible. Constructed as a rich internet application that uses a live-connect technique to communicate with the web server, it offers continuous game play that is not interrupted by interfering web page loads while one is using standard web techniques. First, all participants have to register with a free-to-choose public player’s name. After registration the active game starts. Three successive game phases characterize the active game. During the *Introduction Phase* the learning objectives, principles and procedures of the serious game are presented. The introduction utilizes many in-game pictures to describe possible situations as an example. Additionally, this phase is supported by an educational adviser who explains the learning objectives orally and answers general questions. The game is played in the following *Execution Phase*. In this phase players start to assign appropriate room functionalities, placing interior furniture and equipment onto the ground plan. A player can virtually open his/her medical practice at any time. Opening his/her medical practice starts a simulation. During the simulation virtual agents of the doctor, doctor's assistants, patients and other visitors follow common workflows and use the placed furniture and equipment. If an agent cannot fulfil his/her operation because of a missing requirement a speech bubble appears that describes the requirement in the form of first-person statements. In this situation a player can stop the simulation to plan and integrate a solution to the problem. Players are free to choose the number of repetitions of this enhancement procedure. In the final *Evaluation Phase* a detailed evaluation of the current session is presented. It includes a detailed report of the used furniture and equipment with overall costs and the price of every single item used. Extensive point ranking gives information on which rule accounts for how many points. If a rule did not comply a hint provides possible improvements for the next time. At any time of game play a public player ranking is visible to all players. This ranking shows the relative distance between the players according to their current score. Additionally, the total money spent on interior furnishing and equipment components is presented. The ranking can be projected to a screen to drive competition between the students.

## Results

This section presents results concerning the game design. In-game screenshots show the user interface as well as the game design. In the last part of this section we describe the evaluation results.

### Game play and design

**Figure 3:**
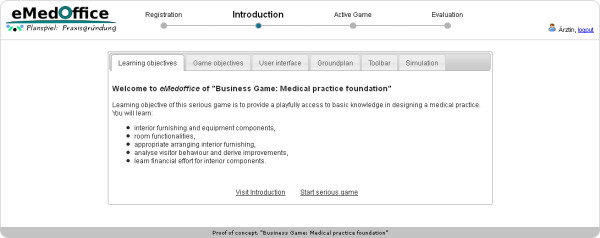
Learning objectives are presented in the introduction screen.

**Figure 4:**
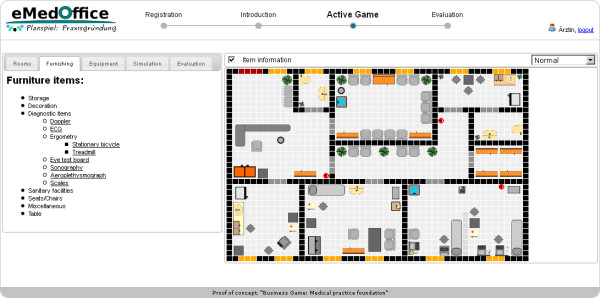
Screenshot: Common game play in an advanced stage of a running game.

**Figure 5:**
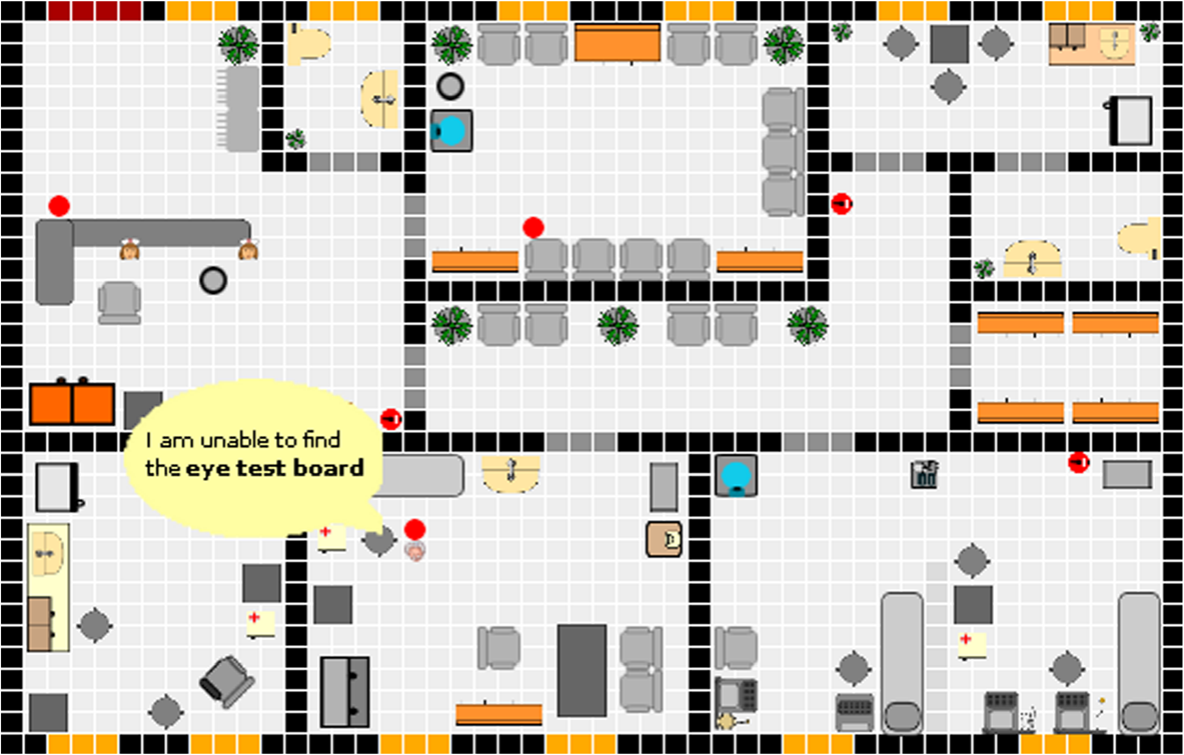
Detail of a screenshot: Simulation phase.

**Figure 6:**
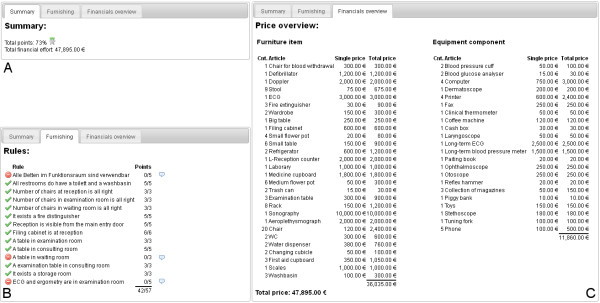
Screenshots: Evaluation phase.

**Figure 7:**
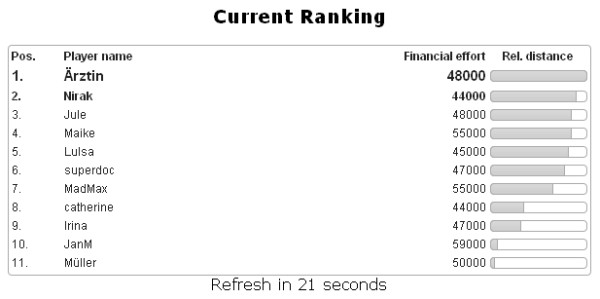
Screenshot: Example of a player ranking.

The game eMedOffice consists of a web-based learning interface and a separate administration user interface. The learner’s user interface (UI) provides access to the game itself. Accordingly, the administration UI supplies tools to manipulate the serious game’s configuration parameters and knowledge base settings and provides a real-time monitor of a running game. Thus, an administrator is aware of players facing difficulties and hence is able to provide individual support by giving specific verbal hints. After registration, the game starts with an introductory screen (Figure 3). The layout of the website is set up to please the eye without compromising usability. At the top left side of the screen is the logo of eMedOffice. At the top middle is a step-wise history bar that indicates which phases are already finished and which will be next. The history bar is intended to provide a general orientation for the user. On the top right side is a login/logout area with the current user’s name. In case of a log-out, a player’s last position in the game is saved and can be retrieved when the player logs in again. In the introduction players have the choice of navigating through the content step-by-step by topic or starting the game without consulting introductory descriptions. Thus, the structure of the introduction supports the various reading and learning habits of players. The first page of the introduction describes the learning objectives. The game starts as soon as the players finish the introduction and switch to the playing phase (Figure 4). In this phase the players assign general functionalities to the rooms, select and place interior furnishings and supplement furniture items with equipment like blood pressure meters or stethoscopes. All these operations can be done with the action bar located on the left side of the working space. The ground plan of the medical practice is located on the right side of the working space. In this example the players have already assigned room functionalities and placed some furniture items on the ground plan. At any time during game play players may decide to open their medical practice. An opening of the medical practice starts a simulation of agents that represent patients, medical doctors and medical doctors’ assistants who use the interior furnishings and interact with each other. When opening a medical practice, players are informed by the game that they can open and close the medical practice as often as they want. A running simulation of randomly selected and concurrent common workflows of a medical practice is depicted in Figure 5. Patients are represented by moving red dots, medical doctor’s assistants are shown by a face wearing a nurse's hat and doctors are represented by a face with white hair. Whenever an agent detects a problem like a missing furniture item or equipment component while executing a workflow-task a speech bubble appears. In this speech bubble the agent uses a first-person statement to report that something is missing or cannot be done. In the case depicted in Figure 6 the doctor’s agent is unable to proceed with an eye test because the eye test board is missing. The players are invited to stop the running simulation and add an eye test board to the examination room. Subsequent to this improvement the players can restart the simulation. When players decide to end game play an evaluation of their result is presented (Figure 6). This evaluation consists of a short summary of the game’s results (Figure 6 a). A detailed view of the rules describes the used scoring system and the results (Figure 6 b). When the players did not comply with a rule a small bubble appears right behind the score. This bubble provides access to a pop-up text providing a hint on how to do better next time. A detailed list of used interior furniture items and equipment component items with single and total prices is provided in the financial overview (Figure 6 c). During game play a public player ranking is projected on a screen visible to all players at all times (Figure 7). The ranking provides information about current rank, financial outlay and distance from the other players according to points achieved. The financial outlay sums all money spent on furniture and equipment items and is presented to the participants, but is not used for ranking or scoring calculations. The public ranking is refreshed every 30 seconds.

### Evaluation results

#### Measurements

**Figure 8:**
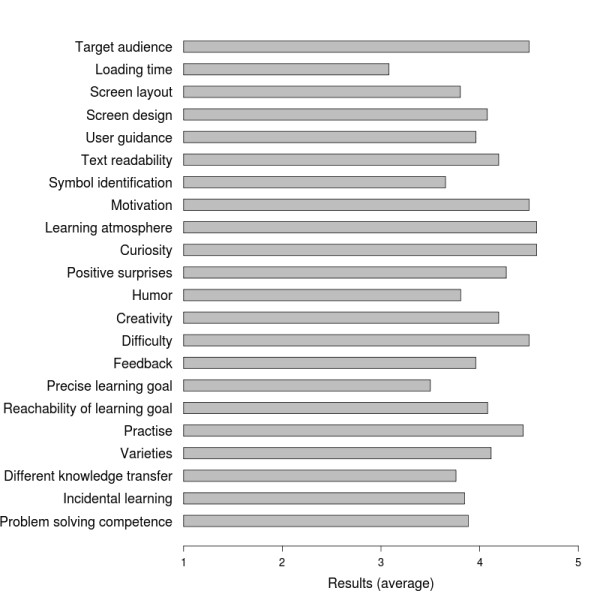
Mean results of usability questionnaire.

In total 41 students (N = 41) participated in the qualification profile and used the proposed game eMedOffice. Analysis of usability aspects of the first intervention (N = 27) revealed high overall usability of the proposed game (4.07 points on average on a scale of one to five) (see Figure 8 for detailed results). For an average reliability (ICC) value we computed 0.91 which indicates that the recorded survey data can be generalized. The second intervention of the evaluation (N = 41) showed indications of the positive effect of the proposed game. A statistical analysis showed a significant difference between the pre and post as well as retro and post groups. This disproves the null hypothesis, revealing a significant difference between pre and post groups as well as retro and post groups. An overview of the computed significance levels between pre, retro and post groups is given in Table 3. It is evident that the mean values of the post groups are higher than those of the pre groups. Based on the results presented in Table 3 the null hypothesis was rejected. Hence we conclude that the players consider the game as a way to improve their abilities and knowledge. This result is an immediate indication of the positive effect of the serious game. Two participants stated that they started the learning course with expert knowledge and finished the training with no knowledge. Moreover, they chose the worst rating without giving any free textual feedback. Thus, we rated the evaluation of those two participants as a general protest and excluded them from our statistical analysis. The protest could have been provoked by the enormous number of course evaluations in which students are asked to take part during a semester.

**Table 3:** Comparison of significances and means of groups

	***pre***			***retro***			***post***		
***St.***	***Sig***_***r***_	***Sig***_***p***_	***m***_***e***_	***Sig***_***e***_	***Sig***_***p***_	***m***_***r***_	***Sig***_***e***_	***Sig***_***r***_	***m***_***p***_
1)	.512	< .001	3.794	.512	< .001	3.714	< .001	< .001	4.512
2)	.225	< .001	3.512	.225	< .001	3.686	< .001	< .001	4.410
3)	1.00	< .001	3.410	1.00	< .001	3.456	< .001	< .001	4.315
4)	.030	< .001	2.282	.030	< .001	2.714	< .001	< .001	3.769
5)	.571	< .001	3.076	.571	< .001	3.294	< .001	< .001	4.156
6)	.023	< .001	2.923	.023	< .001	3.342	< .001	< .001	4.290
7)	.160	< .001	2.948	.160	< .001	3.323	< .001	< .001	4.210

The first column statement (*St.*) shows the index number of the used statements that were introduced in chapter evaluation design. Columns Sig_{e, r, p}_ show computed p-values of the paired t-test for pre (Sig_e_), retro (Sig_r_) and post (Sig_p_) groups. Columns m_{e, r, p}_ show the computed mean values of self-reports according to statements 1-7 (see Table 1).

### Observations

**Figure 9:**
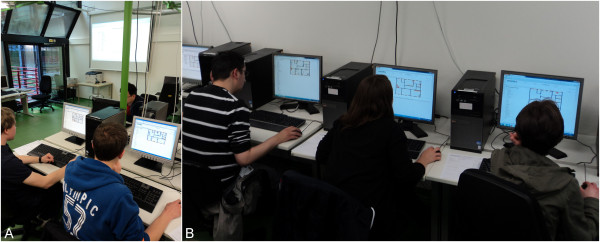
Students playing eMedOffice.

The observations of the educational supervisors revealed that the public ranking strongly encouraged players to communicate with each other and fostered competitive cooperation. One opening question asked very often in player conversations was: ‘What did you do to improve your score?’. Players started to revise their individual solutions critically and initiated active and valuable discussions. Some players compared their individual solutions in detail to find out what made one solution better than another. At the same time players developed strong ambitions to enhance their current solution and some stayed beyond the scheduled time of the course or asked for later access from home to implement further improvements. Thus, in the light of observations by educational supervisors during evaluations of the game it seems to enhance problem-solving competence of the participants in a motivated and collaborative manner. The supervisors observed that the participants repeated the feedback cycle of furnishing the practice, observing the simulation, stopping the simulation and improving the arrangements about three to six times during the course. After debriefing and evaluation, some students asked for more time to enhance their solution. For that reason we made the game available on the internet for attendees of the course, some of whom did continue to use it. Students playing eMedOffice are depicted in Figure 9. Visible to all players, the public ranking is projected with a beamer to a screen on a wall. The public ranking is present in the background of Figure 9 a. The seating arrangement of students is illustrated in Figure 9 b. Students sit in rows and can look at the screen of their immediate neighbours. Those students depicted in Figure 9 b have to turn around to see the public ranking.

## Discussion

**Figure 10:**
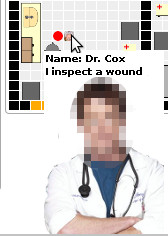
Task information in simulation mode.

By applying ontologies to structure and maintain the knowledge base, we provided a flexible inclusion of different types of rules like necessary distance between objects or required access from a certain direction. Although the initial integration and domain-specific adjustments were complex and time-consuming, a reusable and easily extendable knowledge base was constructed. Object-oriented aspects of ontologies particularly helped to formulate more general rules that applied to a variety of specialized concepts. One example of a general furniture item is a *chair*-concept in contrast to more specific items like a *stool*- or *chair for blood withdrawal*-concept. All instances of a *chair*-concept must provide an accessible seat area which also applies to all inherited concepts. We look forward to reusing these ontologies in other projects that need flexible, extendable and reusable knowledge bases. Using a game to teach interior design of a medical practice revealed some positive aspects. Common processes in a medical practice are visualized and players can analyse and improve the interior design in the light of their findings. When integrating improvements, players are encouraged to explore self-invented ideas. This is supported by the immediate feedback of simulated patients on new furniture and equipment arrangements. Also, players are naturally supported to train for their intended role as a manager of a medical practice: they observe and analyse the behaviour of patients and deduce appropriate actions. According to reports by the educational supervisors of the gaming sessions the public player ranking in particular promoted valuable competitive collaborations and fostered motivated game play. Another motivating aspect of the game was the application of the circle of expertise. The three successive, sustainable and repeated steps of observation, analysis and improvement helped to create a motivating gaming experience throughout the game and helped to increase students' motivation to explore self-invented ideas. With regard to participants who react to stress and need individual support to master competitive situations we plan further investigations of this motivated gaming experience. As a further motivating aspect we integrated amusing features into the game. For example, when players hover over an agent with the mouse in simulation mode, an information box appears that tells the player the name and current action of the agent (Figure 10). Each agent has a unique picture that shows characters from a famous TV show that is currently popular among medical students. According to reports by the educational supervisors these cheerful elements fostered good mood and helped to create an enjoyable learning atmosphere. Some items on the usability questionnaire of the first intervention were less well rated on average in comparison with the rest of the evaluated items. These are (a) Loading time, (b) Precise learning objective and (c) Symbol identification. Shortly after the evaluation the used machines were replaced by new ones which boosted the gaming speed and hence led to more sophisticated game play. Therefore a bad rating for (a) Loading time was compensated in the last evaluation. The rating for (b) Precise learning objective can potentially be increased by a short integrated introductory game play video or a more detailed verbal description of the learning objective and easy accomplishable sub-objectives. The third item (c) Symbol identification could be improved by an offer of more information if a tool in eMedOffice is used incorrectly. Additionally, the introduction could be improved with respect to the in-game symbols. In future, there should be further evaluations that compare eMedOffice with ordinary courses that teach the same topic. Another open challenge is to prove the superiority of eMedOffice in contrast to other teaching methods, a necessary requirement for its enduring integration in courses.

### Future work

Given our experiences in the development and evaluation of eMedOffice, various extensions are possible. One important possible extension is the enhancement of the knowledge base. This includes the formalization of new rules to describe a good working medical practice. More rules would allow the expedient usage of different levels of difficulties and could enable more authentic scoring. Another important aspect is the integration of an internationalisation function that allows players to switch the language of eMedOffice. This is especially important for international courses with many students from foreign countries. Moreover, additional languages could improve the subject-specific foreign languages of all students. An economic circuit could further extend the game to enhance players' commercial and financial understanding. Players could earn virtual money for each successfully treated patient. This earned money could be invested to further enhance the medical practice. In that context it could be an additional motivating aspect if all players competed for the same shared patient pool. Thus, more visits of patients would indicate a ‘better’ medical practice compared with a ‘bad’ one less frequently visited. This would leave more successful players with increasing difficulties and the need to satisfy more varied demands by the patients than less successful players who have more time to evaluate failed workflows. Overall, it could benefit the balance of the game depending on each student’s performance. A shared patient pool could also sensitize players to the growing competition for patients in the real world. Additionally, the evaluation elicited many valuable suggestions from players concerning improvements for the game interaction like an enhanced method to rotate equipment on the ground plan or more detailed information about the placed furniture items.

## Conclusions

The project eMedOffice implements a practical approach for a serious game in a medical environment to teach optimization of design in terms of furnishing, equipment components and furniture items in a general medical practice. Designed as a serious game, it is integrated in the curricular courses of medical study at the RWTH Aachen University Medical School. By using motivating aspects like permanently visible public player ranking and fun elements, the game fostered valuable discussions and competitive collaboration among participants. A statistical analysis of the results of an evaluation provides a clear indication of the positive learning effect of the serious game. A usability questionnaire survey reveals a very good overall score of 4.07 (5=best, 1=worst). In conclusion, we are confident that we can develop even more effective serious games in future that can be integrated into curricular courses of the RWTH Aachen University Medical School.

## Availability and requirements

Project name: eMedOffice

Project home page: http://sourceforge.net/projects/emedoffice/

Operating system(s): Platform independent

Programming language: PHP

Other requirements: Apache2, mod-php, MySQL

License: GNU GPL

Any restrictions to use by non-academics: GNU GPL

## Usability questionnaire

**Figure 11:**
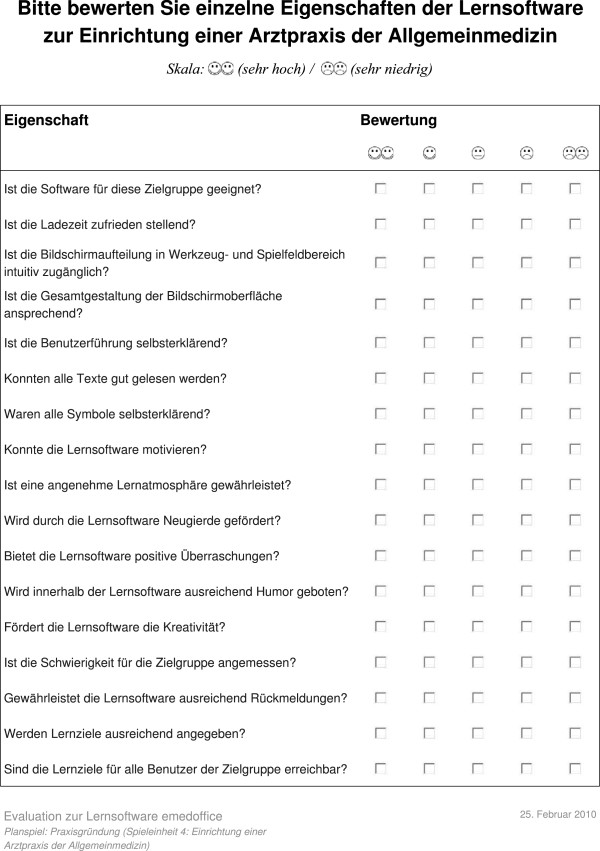
First page of usability questionnaire.

**Figure 12:**
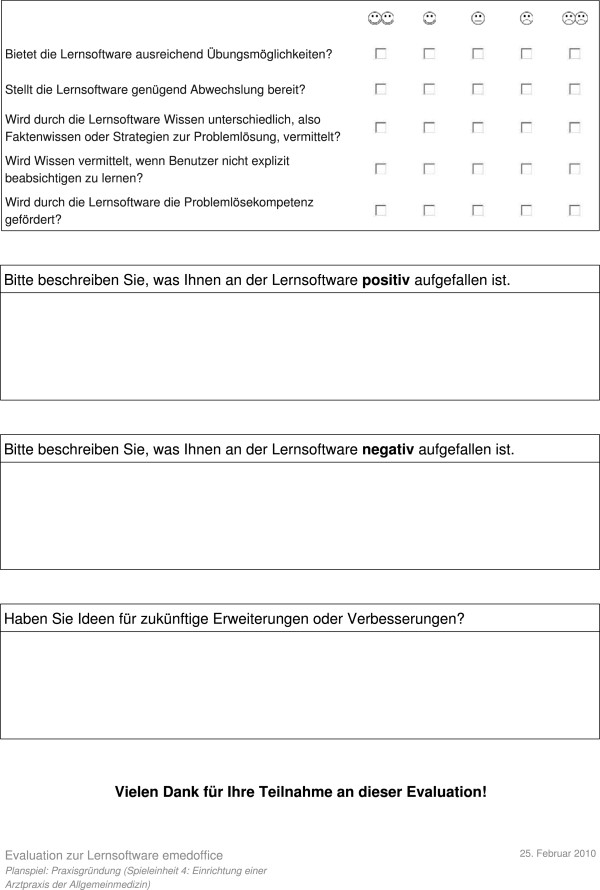
Second page of usability questionnaire.

This section presents the usability questionnaire used to evaluate the quality of the proposed learning game eMedOffice (Figure 11 and Figure 12). As the evaluation took place at the RWTH Aachen Medical School in Germany, all contents are in German.

## Self-report

**Figure 13:**
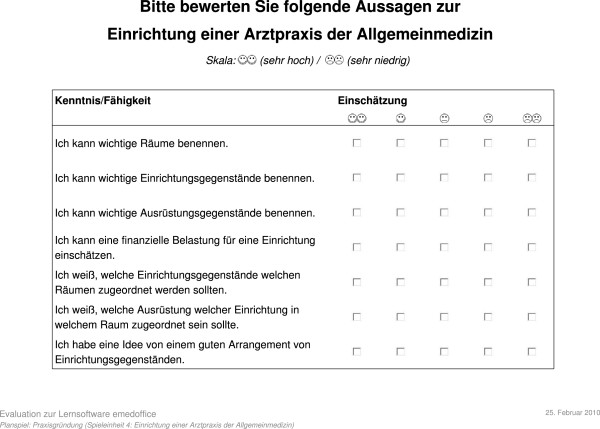
Self-report.

This section provides the self-report sheet that participants had to fill in before and after playing the learning game eMedOffice (Figure 13).

## Competing interests

The authors declare that they have no competing interests.

## Authors’ contributions

AH developed the game eMedOffice and is responsible for the article. NK and MÖ are experts in interior design and helped to create the concept of the game. SJ helped with writing the article. CS supervised the development of the game, performed the evaluations and helped with writing the article. All authors read and approved the final manuscript.

## Pre-publication history

The pre-publication history for this paper can be accessed here:

http://www.biomedcentral.com/1472-6920/12/104/prepub

## References

[B1] Landsberg G Positionspapier „ärztliche versorgung im ländlichen raum DStGB - Deutscher Städte- und Gemeinde 2010 DStGB web site [cited 2011 October 18]. http://www.dstgb.de/dstgb/Kommunalreport/Archiv%202010/%C3%84rztliche%20Versorgung%20im%20l%C3%A4ndlichen%20Raum%20gef%C3%A4hrdet%20-%20Reformans%C3%A4tze%20notwendig/positionspapier_aerztliche_versorgung_1105.pdf

[B2] Hauschild W Klose A Kostenstrukturen bei arzt und zahnarztpraxen 2003 Wirtschaft und Statistik 2003 11 1151 1158 2006

[B3] Lisac M Reimers L Henke KD Schlette S Access and choice–competition under the roof of solidarity in German health care: an analysis of health policy reforms since 2004 Health Econ Policy Law 2010 5 1 31 52 10.1017/S1744133109990144 19732476

[B4] Stock SAK Redaelli M Lauterbach KW Disease management and health care reforms in Germany — does more competition lead to less solidarity? Health Policy 2007 80 1 86 96 10.1016/j.healthpol.2006.02.005 16600418

[B5] Fripp J A future for business simulations? Journal of European Industrial Training 1997 21 4 138 142 10.1108/03090599710171387

[B6] Mayer BW Dale KM Fraccastoro K Moss G Improving transfer of learning: relationship to methods of using business simulation Simulation & Gaming 2010 42 1 64 84 10.1177/1046878110376795

[B7] Hansen MM Versatile, immersive, creative and dynamic virtual 3-D healthcare learning environments: a review of the literature J Med Internet Res 2008 10 3 e26 10.2196/jmir.1051 18762473 PMC2626432

[B8] Adams SA Use of “serious health games” in health care: a review Stud Health Technol Inform 2010 157 160 166 20543383

[B9] Martin A The design and evolution of a simulation/game for teaching information systems development Simulation & Gaming 2000 31 4 445 463 10.1177/104687810003100401

[B10] Garris R Ahlers R Driskell JE Games, motivation, and learning: a research and practice model Simulation & Gaming 2002 33 4 441 467 10.1177/1046878102238607

[B11] Sawyer B Smith P Serious games taxonomy Health San Francisco 2008 [cited 2011 September 19]. http://www.dmill.com/presentations/serious-games-taxonomy-2008.pdf

[B12] Stapleton AJ Serious games: serious opportunities 2004 Australian Game Developers’ Conference, Academic Summit, Melbourne 1 6 [cited 2011 October 2]. http://andrewstapleton.com/wp-content/uploads/2006/12/serious_games_agdc2004.pdf

[B13] Sawyer B From cells to cell processors: the integration of health and video games IEEE Comput Graph Appl 2008 28 6 83 85 10.1109/MCG.2008.114 19004688

[B14] Eck RV Digital game-based learning: It’s Not just the digital natives Who Are restless … Educause Review 2006 41 2 1 16 10.1145/950566.950596

[B15] Susi T Johannesson M Backlund P Serious games – an overview 2007 School of Humanities and Informatics, University of Skövde, Sweden Technical Report HS-IKI-TR-07-001, 2007

[B16] Ritterfeld U Cody MJ Vorderer P Serious games: mechanisms and effects 2009 Taylor & Francis, New York

[B17] Horstmann M Renninger M Hennenlotter J Horstmann CC Stenzl A Blended E-learning in a Web-based virtual hospital: a useful tool for undergraduate education in urology Education for Health 2009 22 2 269 20029750

[B18] Sliney A Murphy D JDoc: a serious game for medical learning First International Conference on Advances in Computer-Human Interaction 2008 39 131 136 10.1109/ACHI.2008.50

[B19] Girardi FM Nieto FB Vitória LP de Borba Vieira PR Guimaráes JB Salvador S Scroferneker ML T- and B-cell ontogeny: an alternative teaching method: T- and B-cell ontogeny game Teach Learn Med 2006 18 3 251 260 10.1207/s15328015tlm1803_11 16776614

[B20] Kanstrup AM Boye N Theory meets practice in the design of e-support for junior registrar doctors Artifact 2007 1 3 190 197 10.1080/17493460701800231

[B21] De Marcos Ortega L Barchino PR Jiménez Rodríguez ML Hilera González JR Martínez Herráiz JJ Gutiérrez De Mesa JA Gutiérrez Martínez JM Otón Tortosa S Using m-learning on nursing courses to improve learning Computers Informatics Nursing CIN 2011 29 5 311 317 10.1097/NCN.0b013e3181fcbddb 21084973

[B22] Crancer J Maury-Hess S Games: an alternative to pedagogical instruction J Nurs Educ 1980 19 3 45 52 6246049

[B23] Gee JP What video games have to teach us about learning and literacy Computers in Entertainment (CIE) 2003 1 1 20 October 2003 10.1145/950566.950595

[B24] Gee JP Learning by design: good video games as learning machines E-learning 2005 2 1 5 16 0.2304/elea.2005.2.1.5

[B25] Gruber TR Toward principles for the design of ontologies used for knowledge sharing International Journal of Human Computer Studies 1995 43 5–6 907 928 10.1006/ijhc.1995.1081

[B26] Noy N Sintek M Decker S Creating semantic web contents with protege-2000 IEEE Intelligent Systems 2001 16 2 60 71 10.1109/5254.920601

[B27] Hart PE Nilsson NJ Raphael B A formal basis for the heuristic determination of minimum cost paths IEEE Transactions on Systems Science and Cybernetics 1968 4 2 100 107 10.1109/TSSC.1968.300136

[B28] Holzinger A Beurteilungskriterien für lernsoftware 2003 [cited 2011 September 20]. http://user.meduni-graz.at/andreas.holzinger/holzinger%20de/papers%20de/Beurteilung_Lernsoftware.pdf

[B29] Ulrich M Komplexität anpacken: Mit planspielen erfolgreiches handeln erlernen 2006 Werner-und-Elisabeth-Kollath-Stiftung, Tagungsband zur 7. Werner-Kollath-Tagung, Universität Giessen [cited 2011 September 10]. http://www.ucs.ch/service/download/docs/artikelkomplexitaetplanspiele.pdf

